# Farm‐Level Factors Associated With Multidrug‐Resistant *Escherichia coli* and *Klebsiella* spp. in Bulk Tank Milk in Tigray, Northern Ethiopia

**DOI:** 10.1002/mbo3.70265

**Published:** 2026-03-16

**Authors:** Goyitom Gebremedhn Gebru, Saravanan Muthupandian, Enquebaher Kassaye

**Affiliations:** ^1^ Department of Medical Microbiology Tigray Health Research Institute Mekelle Tigray Ethiopia; ^2^ Department of Veterinary Public Health and Food Safety College of Veterinary Sciences Mekelle University Mekelle Tigray Ethiopia; ^3^ Department of Medical Laboratory Technology, Faculty of Applied Medical Sciences University of Tabuk Tabuk Saudi Arabia; ^4^ Prince Fahad bin Sultan Chair for Biomedical Research University of Tabuk Tabuk Saudi Arabia

**Keywords:** dairy farms, *Escherichia coli*, Ethiopia, food safety, *Klebsiella* spp., multidrug resistance, raw milk

## Abstract

Raw milk is widely consumed unpasteurized in Ethiopia, posing food safety risks when contaminated with multidrug‐resistant (MDR) bacteria. This study investigated farm‐level factors associated with the occurrence of MDR *Escherichia coli* and *Klebsiella* spp. in bulk tank raw milk from dairy farms in the Tigray region, Northern Ethiopia. A cross‐sectional study was conducted among 178 dairy farms, combining microbiological analysis of bulk tank milk with data on farm hygiene, animal health, and antimicrobial use (AMU) practices. A total of 119 isolates (70 *E. coli* and 49 *Klebsiella* spp.) were recovered, of which 32.8% were classified as MDR. Multivariable analysis showed that infrequent washing of milking utensils (once daily or less) was strongly associated with MDR‐positive milk (adjusted odds ratio [AORs] = 7.41; 95% CI, 1.94–28.31). Self‐administration of antimicrobials by farm owners (AOR = 3.73; 95% CI, 1.48–9.39) and a history of mastitis in the herd (AOR = 4.79; 95% CI, 1.39–16.52) were also independently associated with MDR occurrence, while herd size was not significantly associated. The presence of MDR *E. coli* and *Klebsiella* spp. in raw milk represents a significant food safety concern in smallholder dairy systems. Targeted interventions focusing on improved milking hygiene, responsible AMU under veterinary supervision, and effective mastitis management are essential to reduce the entry of resistant bacteria into the dairy value chain and limit consumer exposure.

## Graphical abstract

This cross‐sectional study identified farm‐level factors associated with multidrug‐resistant (MDR) *Escherichia coli* and *Klebsiella* spp. in bulk tank milk in Tigray, Northern Ethiopia, emphasizing the role of antimicrobial use practices and hygiene management in MDR occurrence within dairy production systems.

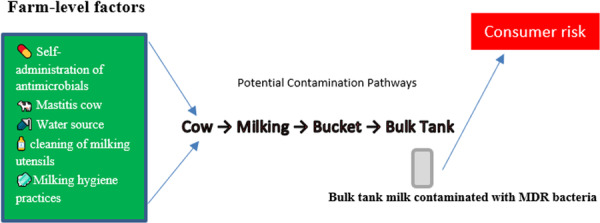

**Abbreviations**
AMR: antimicrobial resistant
AOR: adjusted odds ratio
ATCC: American Type Culture Collection
CI: confidence interval
CLSI: Clinical and Laboratory Standards Institute
EPHI: Ethiopian Public Health Institute
*E. coli*
: *Escherichia coli*
*K. oxytoca*
: *Klebsiella oxytoca*
*K. pneumoniae*
: *Klebsiella pneumoniae*
MDR: multidrug resistant
MHA: Muller–Hinton agar
ODK: open data kit
OR: odds ratio
SPSS: Statistical Package for the Social Sciences
THRI: Tigray Health Research Institute
WHO: World Health Organization

## 1 Introduction

Milk is a highly nutritious food that plays an essential role in human nutrition and livelihoods worldwide, providing essential proteins, fats, vitamins, and minerals (Sanjulián et al. 2025). However, its rich nutrient composition makes it an excellent medium for microbial growth (Moatsou and Moschopoulou 2014), and when mishandled, it can easily become contaminated and serve as a vehicle for transmitting harmful microorganisms to consumers (Oliver et al. 2005). In many developing countries, including Ethiopia, raw milk is often sold and consumed unpasteurized, which can facilitate the transmission of foodborne pathogens (Gume et al. 2023). This poses serious food safety and public health risks, especially in communities where milk is consumed daily by children, pregnant women, and the elderly.

Among the bacteria that commonly contaminate milk, *Escherichia coli* and *Klebsiella* species are of particular importance. These bacteria are common inhabitants of the intestinal tracts of humans and animals, but can contaminate milk through fecal material, contaminated water, milk containers, or unhygienic milking practices (Martin et al. 2016; Mesele et al. 2023; Sarba et al. 2023). The presence of these bacteria in raw milk not only indicates poor hygienic practices but also serves as a proxy indicator of fecal contamination and potential zoonotic transmission (Mpatswenumugabo et al. 2023). Beyond their role as indicators, both pathogens are also capable of causing serious infections in both animals and humans.

In recent years, the emergence of antimicrobial‐resistant (AMR) bacteria in foods of animal origin has become a major global health threat. *E. coli* and *Klebsiella* spp. can serve as reservoirs and disseminators of resistance genes, including those encoding extended‐spectrum beta‐lactamases (ESBLs), in milk and dairy environments (Gelalcha et al. 2023; Homeier‐Bachmann et al. 2022; Youssef et al. 2025). The detection of such resistant strains in milk is alarming, as raw milk is commonly consumed unpasteurized in Ethiopia, increasing the risk of transmitting resistant pathogens to humans (Gume et al. 2023).

The dairy sector in Ethiopia, including the Tigray region, operates largely under smallholder conditions characterized by limited access to veterinary services, water scarcity, inappropriate antimicrobial use (AMU), and poor hygiene practices during milking and milk handling (Deddefo et al. 2023; Feyisa et al. 2024; Kebede et al. 2025; Lencho and Seblewongel 2018). These conditions may favor the introduction and persistence of resistant bacteria in milk and the farm environment. However, despite several reports from other parts of Ethiopia (Beyene et al. 2024; Mesele 2018; Mesele et al. 2023), there is limited information from the Tigray region on the farm hygiene practices, AMU behaviors, and animal health conditions, such as mastitis, that may influence the introduction, emergence, and persistence of multidrug‐resistant (MDR) bacteria in dairy farm environments.

Therefore, the current study aims to determine the farm‐level risk factors associated with the occurrence of MDR *E. coli* and *Klebsiella* spp. isolates in raw bulk tank milk from dairy farms in the Tigray region, northern Ethiopia. The findings will provide important evidence to enhance dairy hygiene practices, support antimicrobial stewardship, and strengthen food safety interventions to reduce the spread of resistant pathogens through the milk value chain.

## 2 Materials and Methods

### 2.1 Study Design and Setting

A cross‐sectional study was conducted from December 2024 to May 2025 in three selected zones of the Tigray region: Eastern, Southeastern, and Mekelle. Five woredas or subcities were purposively selected from these zones. The selected areas included Ayder, Hadnet, and Hawelti from Mekelle; Agulae from the Eastern Zone, and Hagereselam from the Southeastern Zone. Mekelle was selected as the primary urban site due to its status as the capital city of the Tigray region, which has a large population and high milk consumption. The other two study sites, Agulae and Hagereselam, were selected for their proximity to Mekelle and the high concentration of dairy farms that supply raw milk to the large population living in Mekelle city. The purposive selection is intended to capture representative data from both the major consumption area and the primary milk production zones, representing the dairy value chain in the region.

### 2.2 Sample Size and Sampling Technique

This study utilizes the milk‐specific data set generated by a broader study, and the sample size was calculated as described in that previous study (Gebru et al. 2025). Briefly, our previous study aimed to detect *E. coli* and *Klebsiella* spp. in dairy farm environments.

For this risk factor analysis, we used all the farms with positive results for *E. coli* or *Klebsiella* spp. in the bulk tank milk, creating a linked data set of microbiological outcomes and farm management variables for regression modeling.

The calculated sample size was 196 farms. However, of the 196 randomly selected farms, 18 did not participate due to refusal (*n* = 5), temporary farm closure (*n* = 7), or herd absence during the sampling period (*n* = 4). The final sample consisted of 178 farms (response rate 90.8%).

Sample size was allocated proportionally to the sizes of dairy farms in the selected study areas. The number of dairy farms in the selected districts with the respective proportional sample size allocated is listed in Table 1. A simple random sampling technique was used to collect the sample from the selected sites.

**Table 1:** Sampling frame and distribution of calculated and collected sample sizes across selected districts in Tigray Region, Northern Ethiopia.

S. No.	Districts	Number of dairy farms	Calculated sample size	Collected sample
1	Hadnet	290	41	36
2	Hawelti	260	38	32
3	Ayer	302	43	38
4	Hagereselam	258	37	36
5	Agulae	259	37	36
Total		1379	196	178

### 2.3 Sample Collection

Approximately 30 mL of milk was aseptically collected from each bulk tank milk (pooled milk from the entire lactating herd) into sterile screw‐cap containers. All containers were prelabeled with a unique sample code, farm identifier, and date of collection. Samples were immediately placed in insulated cool boxes and transported to the Microbiology Laboratory of the Tigray Health Research Institute (THRI) within 4 h of collection to maintain sample integrity.

#### 2.3.1 Isolation and Identification of *E. coli* and *Klebsiella* spp.

Isolation and identification procedures followed standard bacteriological techniques as described in the Bacteriological Analytical Manual (FDA 2020; Quinn et al. 2011). In brief, 25 mL of each milk sample was homogenized in 225 mL of buffered peptone water for pre‐enrichment. After incubation, a loopful of the enriched suspension was streaked onto MacConkey agar (Oxoid Ltd., UK) and incubated at 37°C for 24–48 h.

Presumptive colonies were selected based on lactose fermentation characteristics, colony morphology, and Gram staining. Selected colonies were subcultured onto Tryptic Soy Agar to obtain pure cultures. Final identification was performed using a panel of biochemical tests, including Triple Sugar Iron reaction, indole production, citrate utilization, urease activity, and motility.

### 2.4 Antimicrobial Susceptibility Testing

The Kirby‐Bauer disk diffusion method was used to test susceptibility to antimicrobials, following the guidelines set by the Clinical and Laboratory Standards Institute (CLSI 2024). A sterile wire loop was used to take pure colonies from fresh cultures. These colonies were then emulsified in 0.85% sterile normal saline until the resulting turbidity matched a 0.5 McFarland standard. This bacterial suspension was then used to inoculate the surface of Muller–Hinton agar (HiMEDIA, India) using the lawn culture technique.

Thirteen antimicrobial disks representing major drug classes were tested: Amoxicillin‐Clavulanic acid (20/10 µg), Piperacillin‐Tazobactam (110 µg), Ampicillin‐Sulbactam (30 µg), Cefotaxime (30 µg), Ceftazidime (30 µg), Ceftriaxone (30 µg), Cefoxitin (30 µg), Aztreonam (30 µg), Amikacin (30 µg), Meropenem (10 µg), Tetracycline (30 µg), Ciprofloxacin (5 µg), and Sulfamethoxazole‐Trimethoprim (23.75/1.25 µg). The plates were incubated at 37°C for 16–18 h, after which inhibition zone diameters were measured and interpreted using CLSI (2024). MDR was defined as resistance to one or more antimicrobial agents in three or more different antimicrobial classes (Magiorakos et al. 2012).

### 2.5 Quality Assurance

Quality control strains (*E. coli* American Type Culture Collection [ATCC] 25922 and *Klebsiella pneumoniae* ATCC 700603), obtained from the Ethiopian Public Health Institute, were used to evaluate the performance of culture media, the accuracy of biochemical tests, and the potency of antimicrobial disks.

### 2.6 Questionnaire Survey and Risk Factor Assessment

A structured questionnaire was administered to dairy farm owners or milkers to collect information on potential farm‐level determinants associated with MDR (*E. coli* and *Klebsiella* spp.) isolates in bulk tank milk. Data were collected using the electronic data collection tool, Open Data Kit Collect on Android devices. Responses were cross‐checked with on‐site observations to ensure data accuracy.

The questionnaire included information on the following categories: sociodemographic factors, milking and general hygienic practices, animal health and AMU, such as the history of antibiotic use, whether the farmer self‐treats cows, and compliance with the milk withdrawal period.

### 2.7 Data Processing and Analysis

Laboratory and questionnaire data were entered into Microsoft Excel and analyzed using Statistical Package for the Social Sciences version 26. Descriptive statistics were used to summarize the sociodemographic and farm management characteristics. The association between the potential risk factors (independent variables) and the occurrence of MDR *E. coli* and/or *Klebsiella* isolates in milk was assessed using binary logistic regression. For the risk factor analysis, MDR status was defined at the farm level. A dairy farm was classified as MDR‐positive if at least one MDR *E. coli* or *Klebsiella* isolate was detected in its bulk tank milk sample, whereas farms with no MDR isolates were classified as MDR‐negative. This farm‐level binary outcome variable (MDR: Yes/No) was then used in the logistic regression models to assess associations with farm management, hygiene, and AMU practices. Univariate analysis was conducted initially, and variables with *p* < 0.25 were selected for inclusion in the final multivariable logistic regression models. Adjusted odds ratios (AORs) were computed to identify independent predictors. A *p* < 0.05 was considered statistically significant for the final models.

### 2.8 Ethical Considerations

Ethical approval for this study was obtained from the Institutional Review Board of THRI (Ref. No. THRI/59/2024). Verbal consent was obtained from all farm owners or milkers before data and sample collection, and all information was handled confidentially.

## 3 Results

### 3.1 Sociodemographic and Farm Characteristics

A total of 178 dairy farms were included in this study. Among the dairy farms, 135 (75.8%) were owned by males, and nearly half (48.9%) were within the age group of 45–64 years, followed by those aged 25–44 years (37.1%). Regarding educational status, primary school education was the most common level attained by owners (38.2%). A smaller proportion had diploma‐level or higher education (15.7%), and 15.7% were unable to read and write. The majority of the dairy farms, 170 (95.5), were smallholder dairy farms, and all of which practiced intensive farming (Table 2).

**Table 2:** Sociodemographic and farm characteristics of the study farms in the Tigray region, Northern Ethiopia (December 2024–May 2025).

Variable	Category	Frequency (*n* = 178)	Percent (%)
Owner gender	Male	135	75.8
	Female	43	24.2
Owner age in years	15–24	9	5.1
	25–44	66	37.1
	45–64	87	48.9
	65+	16	9.0
Owner's level of education	Unable to read and write	28	15.7
	No formal school, but able to read and write	13	7.3
	Primary school	68	38.2
	Secondary school	41	23.0
	Diploma and above	28	15.7
Owner's years of service on the farm	≤ 5	20	11.2
	> 5	158	88.8
Herd size (number of milk cows)	Small	170	95.5
	Large	8	4.5
Farm type	Intensive	178	100.0
Milker gender	Male	147	82.6
	Female	31	17.4
Milker's age in years	15–24	78	43.8
	25–44	55	30.9
	45–64	37	20.8
	65+	8	4.5
Milker's level of education	Unable to read and write	38	21.3
	No formal school, but able to read and write	6	3.4
	Primary school	75	42.1
	Secondary school	44	24.7
	Diploma and above	15	8.4

Most milkers were male (82.6%), with the largest proportion aged 15–24 years (39.9%), followed by those aged 25–44 years (30.9%). A small proportion were younger than 15 years (3.9%) or aged 65 years and above (4.5%). Educational status of milkers was generally low, with 42.1% having primary education, 24.7% secondary education, and 21.3% unable to read or write, while only 8.4% had diploma‐level education or above.

### 3.2 Multidrug Resistance Among *E. coli* and *Klebsiella* Isolates

Among the 178 bulk tank milk samples, a total of 119 *E. coli* and *Klebsiella* spp. were isolated from the raw bulk tank milk samples, of which 39 (32.8%) were MDR (Table 3). In our study, all dairy farms found to be positive for MDR had only a single MDR isolate (either *E. coli o*r *Klebsiella* spp.). This MDR status was used as the binary outcome variable (MDR Yes/No) in the logistic regression model to identify the farm‐level risk factors associated with the occurrence of the MDR isolates.

**Table 3:** Distribution of multidrug resistance among *Escherichia coli* and *Klebsiella* spp. in raw bulk tank milk from dairy farms in the Tigray region, Northern Ethiopia (December 2024–May 2025).

Bacterial isolates	Total isolates	MDR isolates	MDR (%)
*E. coli*	70	22	31.4
*Klebsiella pneumoniae*	22	7	31.8
*Klebsiella oxytoca*	27	10	37.0
Total	119	39	32.8

*Note:* Detailed antimicrobial resistance profile of the isolates is described in a previously published article (Gebru et al. 2025).

Abbreviation: MDR, multidrug resistant.

### 3.3 Farm‐Level Risk Factors Associated With the Occurrence of MDR *E. coli* and *Klebsiella* spp.

To identify farm management, hygienic practices, and AMU‐related factors associated with *MDR E. coli* and *Klebsiella* spp. contamination of milk, a bivariate logistic regression analysis was conducted, as shown in Table 4. All independent variables were screened in the initial bivariate logistic regression analysis to select those with *p* < 0.25 for inclusion in the multivariate logistic regression analysis. The variables that met this criterion include herd size, frequency of washing milking utensils, self‐administration of antimicrobials by the owner, compliance with the withdrawal period, and a history of mastitis in cows. However, to avoid model overfitting, only variables with epidemiological relevance and meeting the events‐per‐variable assumption were retained in the final multivariable model.

**Table 4:** Bivariate analysis of farm‐level factors associated with MDR *Escherichia coli* and *Klebsiella* contamination of bulk tank milk in dairy farms (*n* = 119) in the Tigray region, Northern Ethiopia (December 2024–May 2025).

Variable	Category	MDR‐positive farms, *n* (%)	COR (95% CI)	*p* value
Sex of dairy farm owner	Male	31 (33.0)	1.05 (0.41–2.70)	0.933
	Female	8 (32.0)	1.00	Reference
Age of dairy farm owner (years)	≤ 44	13 (31.7)	0.81 (0.20–3.27)	0.767
	≥ 45	26 (36.1)	1.00	Reference
Educational level of the farm owner	≤ Primary	23 (32.4)	1.06 (0.45–2.52)	0.891
	≥ Secondary	16 (33.3)	1.00	Reference
Owner's experience in dairy farming	≤ 5 years	5 (33.3)	1.03 (0.33–3.25)	0.966
	> 5 years	34 (32.7)	1.00	Reference
Herd size	Large	4 (57.1)	2.933 (0.62–13.81)	0.173
	Small	35 (31.3)	1.00	Reference
Frequency of washing milking utensils	Once/day or less	11 (73.3)	7.37 (2.17–25.05)	0.001^a^
	≥ Twice/day	28 (27.2)	1.00	Reference
Self‐administration of antimicrobials by the owner	Yes	19 (50.0)	3.050 (1.354–6.870)	0.007^a^
	No	20 (24.7)	1.00	Reference
Compliance with the withdrawal period	No	30 (28.6)	0.22 (0.07–0.72)	0.012^a^
	Yes	9 (64.3)	1.00	Reference
History of mastitis in cows	Yes	34 (39.1)	3.46 (1.22–9.87)	0.020^a^
	No	5 (15.6)	1.00	Reference

*Note:* Percentages represent the proportion of MDR‐positive farms within each category.

Abbreviations: CI, confidence interval; COR, crude odds ratio; MDR, multidrug resistant.

^a^ Significantly associated.

In the multivariable logistic regression analysis, factors independently associated with MDR *E. coli* and *Klebsiella* spp. contamination of bulk tank milk is presented in Table 5. After adjustment, farms that washed milking utensils once per day or less had significantly higher odds of MDR contamination compared with those washing at least twice per day (AOR = 7.405; 95% CI, 1.937–28.305). Self‐administration of antimicrobials by dairy farm owners was strongly associated with MDR occurrence (AOR = 3.729; 95% CI, 1.482–9.388). In addition, farms with a history of mastitis in cows had increased odds of MDR contamination (AOR = 4.789; 95% CI, 1.389–16.519). Herd size was not independently associated with MDR after adjustment (*p* > 0.05).

**Table 5:** Multivariate analysis of farm‐level factors associated with multidrug‐resistant *Escherichia coli* and *Klebsiella* contamination of bulk tank milk in dairy farms (*n* = 119) in the Tigray region, Northern Ethiopia (December 2024–May 2025).

Variable	Category	AOR	(95% CI)	*p* value
Herd size	Small	1.00	Reference	
	Large	1.802	0.287–11.317	0.530
Frequency of washing milking utensils	Once/day or less	7.405	1.937–28.305	0.003^a^
	≥ Twice/day	1.00	Reference	
Self‐administration of antimicrobials by the owner	Yes	3.729	1.482–9.388	0.005^a^
	No	1.00	Reference	
History of mastitis in cows	Yes	4.789	1.389–16.519	0.013^a^
	No	1.00	Reference	

Abbreviations: AOR, Adjusted Odds Ratio; CI, Confidence Interval.

^a^ Significantly associated.

## 4 Discussion

This study identified key farm‐level factors associated with the occurrence of MDR *E. coli* and *Klebsiella* spp. in raw bulk tank milk from dairy farms in the Tigray region. Understanding how these factors interact is essential for interpreting the food safety risks within smallholder dairy systems.

The proportion of MDR isolates observed in this study (32.8%) is comparable to reports from other parts of Ethiopia (Belayneh et al. 2025; Genet et al. 2025) and several low‐ and middle‐income countries, including Kenya (Byarugaba et al. 2024; Sombie et al. 2022), Uganda (Byarugaba et al. 2024), India (Jindal et al. 2021), and parts of Southeast Asia (Yang et al. 2021). In contrast, substantially lower prevalence has been documented in Europe and North America (Drugea et al. 2025; Tartor et al. 2021). Stronger regulatory systems, routine pasteurization, better hygiene infrastructure, and stricter control of AMU in high‐income settings likely explain these regional differences.

The detection of MDR bacteria in raw milk has direct food safety implications (Mwasinga et al. 2023; Oliver et al. 2005). Raw milk is commonly consumed in our study area without pasteurization. The presence of MDR *E. coli* and *Klebsiella*, therefore, presents a direct risk to consumers (Gelalcha et al. 2023; Mwasinga et al. 2023). Infections caused by such bacteria are harder to treat and may lead to prolonged illness. Moreover, milk can act as a vehicle for transferring resistance genes to the human gut microbiota, contributing to the wider spread of AMR in the community (Mangroliya et al. 2025; World Health Organization 2024; Tóth et al. 2020).

In dairy farms, poor hygiene practices significantly contributed to MDR bacteria contamination of milk (Asfaw et al. 2023; Du et al. 2020). In this study, farms that washed milking utensils once per day or less had significantly higher odds of MDR‐positive milk as compared with those that washed twice or more frequently. This finding is consistent with studies from Ethiopia (Belayneh et al. 2025; Torka 2024), Nepal (Rame Hau et al. 2025), and Pakistan (Ahmad et al. 2021). Infrequently cleaned utensils allow milk residues to build up, creating conditions that support bacterial survival and biofilm formation (Briega et al. 2025; Chowdhury et al. 2025). Biofilms, in turn, can protect bacteria from cleaning agents and promote the persistence of resistant strains within the dairy environment (Ostrov et al. 2019).

In addition to hygiene, AMU practices were a major driver of MDR occurrence (Penati et al. 2024; Ramandinianto et al. 2020). Our study found a strong association between dairy farm owners' self‐administration of antimicrobials and MDR *E. coli* or *Klebsiella* spp. contamination. Similar findings have been reported in Ethiopia (Belayneh et al. 2025), Nigeria (Aworh et al. 2022), and India(Jindal et al. 2021). The use of antibiotics without veterinary guidance often involves inappropriate drug selection, incorrect dosing, or unnecessary treatment (Alhaji and Isola 2018; Gemeda et al. 2020). These practices exert strong selective pressure, favoring the emergence and maintenance of resistant bacteria in dairy herds.

Animal health status also has a significant contribution to milk contamination with MDR bacteria (Belayneh et al. 2025; Manimaran et al. 2025). In this study, farms with a history of mastitis in cows had higher odds of MDR contamination. Mastitis commonly necessitates repeated antimicrobial treatment, often with broad‐spectrum agents (Konishi et al. 2023; McDougall et al. 2022; Rana et al. 2022). Studies from Ethiopia, Egypt, and Brazil have shown that mastitic cows can shed resistant bacteria into milk, leading to contamination of bulk tanks (Abed et al. 2021; Alves et al. 2024; Balemi et al. 2021). Inadequate diagnosis and lack of targeted therapy may further amplify this problem.

In this study, herd size was not independently associated with MDR occurrence. This suggests that management quality and AMU behaviors are more important than farm size alone (Smith et al. 2023; Vijay et al. 2025; Zhang et al. 2022). Similar observations have been reported in other smallholder dairy systems, where even small farms pose substantial food safety risks when hygiene and antimicrobial practices are suboptimal (Nyokabi et al. 2024; Nyokabi et al. 2021).

Despite these important findings, there are a few limitations in our study, including the cross‐sectional study design, which limits causal inference between farm‐level practices and the occurrence of MDR bacteria in milk. In addition, AMU and management data were partly based on self‐reported information, which may be subject to recall or reporting bias. The absence of detailed data on animal sourcing and quarantine practices limits the ability to distinguish between imported and farm‐maintained resistance. Nevertheless, the study provides useful farm‐level evidence from a data‐limited region and underscores the need for targeted hygiene improvement, responsible AMU, and enhanced mastitis management to mitigate the risk of MDR bacteria entering the milk value chain. Furthermore, future longitudinal studies incorporating herd replacement dynamics would provide deeper insight into resistance introduction pathways.

## 5 Conclusion

This study highlights a concerning level of MDR *E. coli* and *Klebsiella* spp. in raw milk from dairy farms in Tigray. Their presence poses a direct risk to consumers in a region where unpasteurized milk is commonly consumed. Our findings show the critical role of farm‐level management practices in shaping AMR risks within smallholder dairy systems. Therefore, strengthening hygiene practices, promoting responsible AMU under veterinary guidance, and improving mastitis prevention and management should be prioritized to reduce the burden of MDR bacteria along the milk value chain. Furthermore, implementing integrated food safety and antimicrobial stewardship interventions within a One Health framework is essential to safeguard public health.

## Author Contributions

**Goyitom Gebremedhn Gebru:** conceptualization, methodology, formal analysis, investigation, writing – original draft, writing – review and editing, visualization. **Saravanan Muthupandian:** writing – review and editing, visualization, supervision. **Enquebaher Kassaye:** writing – review and editing, visualization, supervision.

## Funding

The authors received no specific funding for this work.

## Ethics Statement

Ethical approval for this study was obtained from the Institutional Review Board of Tigray Health Research Institute (Refs No. THRI/59/2024).

## Conflicts of Interest

The authors declare no conflicts of interest.

## Data Availability

The data sets used and/or analyzed during the current study are available from the corresponding author on reasonable request.
